# An Atypical Presentation of a Unicameral Bone Cyst in the Left Humerus: A Case Report

**DOI:** 10.7759/cureus.64679

**Published:** 2024-07-16

**Authors:** Priyansh Sahu, Suhas Tivaskar, Saurabh Somankar, Sharwani Sonewane, Anurag Luharia

**Affiliations:** 1 Department of Radiology, Datta Meghe Institute of Higher Education and Research, Wardha, IND

**Keywords:** orthopedic, benign tumors, x-ray, mri, simple bone cyst, proximal humerus

## Abstract

Simple bone cysts (SBCs) are the most common osteolytic lesions in children, often leading to pathological fractures of non-weight-bearing bones. These benign tumors primarily arise in the proximal humerus, femur, or calcaneus. The cystic cavity is filled with serous or serosanguineous fluid and lined by a thin fibrovascular connective tissue membrane. The etiological factors include disturbances in bone growth, local venous obstruction, synovial origin disorders, and genetic predispositions. SBCs are most frequently observed in individuals from birth to 20 years of age. The report presented a case of a 12-year-old male patient who was admitted to the hospital with a history of a mass on his left upper arm. The mass had an acute onset and gradually progressed to its current size over three to four weeks, after which it became nonprogressive. The patient had been healthy until the age of four, after which he experienced frequent fractures of the left arm following trivial trauma. Multiple traumas led to the gradual formation of a diffuse mass over the upper part of the humerus. Radiological imaging modalities, such as X-ray and magnetic resonance imaging, are crucial in diagnosing bone cysts and evaluating their clinical conditions. Treatment can involve the injection of bone marrow or steroids into the cyst to facilitate the healing process.

## Introduction

Simple bone cysts (SBCs) are considered benign conditions in children and are typically located in the proximal humeral metaphysis. Patients with these cysts often experience a high frequency of pathological fractures and recurrences throughout the course of the condition. The underlying cause of these conditions, whether due to surgical treatment, fractures, or steroid injections, remains unclear [1]. Although steroid injections can offer a nonoperative treatment option, multiple procedures are sometimes necessary to achieve healing. In such cases, open curettage accompanied by bone grafting remains the cornerstone of treatment and is superior to steroid injections [2].

Orthopedic surgeons are usually consulted when cysts are detected on radiographs taken during childhood, often following a pathological fracture. Radiological examinations typically reveal lesions that are concentrically located with clear boundaries, presenting as monocular or multifocal lytic lesions that are slightly expanded. Since the lesion is in a non-weight-bearing part of the humerus, there is a significant risk of pathological fractures [3]. While all SBCs will eventually heal spontaneously, they will not completely subside until the bone matures, during which time movement is also restricted. Determining the appropriate timing for surgery and ensuring the best prognosis for the patient are crucial aspects of managing children with SBCs [4].

## Case presentation

Patient information

The report presented a 12-year-old male patient who arrived at the hospital medicine department with a chief complaint of a mass on his left upper arm, which had persisted for the past year. He exhibited no symptoms of pain, swelling, or restriction of movement. The patient had a history of three previous fractures at the same location on his left upper arm.

Medical/surgical history

The patient had experienced three fractures at the same spot in the left upper arm. According to the patient's history, the first fracture occurred in 2020 after slipping, resulting in pain and a restricted range of motion at the shoulder joint. The second fracture happened in 2022 after a fall from a staircase, and the third fracture occurred in 2023 after another slip. There was no history of persistent pain, local rise in temperature, or restriction of movement. Despite these fractures, the patient could lift 10 kg of weight with his left upper arm.

Physical examination/investigations

The procedure began with a physical examination. The patient had a normal pulse rate of 84 beats per minute, a respiratory rate of 16 breaths per minute, and a blood pressure of 110/70 mmHg. His body mass index was 16.3 kg/m^2^, height was 148 cm, and weight was 40 kg. There were no signs of pallor, clubbing, cyanosis, or edema in the feet. The following parameters were reported during the physical examination, as illustrated in Table 1.

**Table 1:** Physical examination parameters

S. no.	Examination	Status
1	General examination	Normal
2	Nutrition	Normal
3	Hairs	Normal
4	Eyes and sclera	Normal
5	Ears	Normal
6	Tongue	Normal
7	Teeth	Normal
8	Pupils	B/L reactive to lights
9	Respiratory system	B/L air entry present, no added sounds
10	Cardiovascular system	S1 and S2, no murmur
11	Central nervous system	The patient is conscious and oriented to place and person

**Figure 1:**
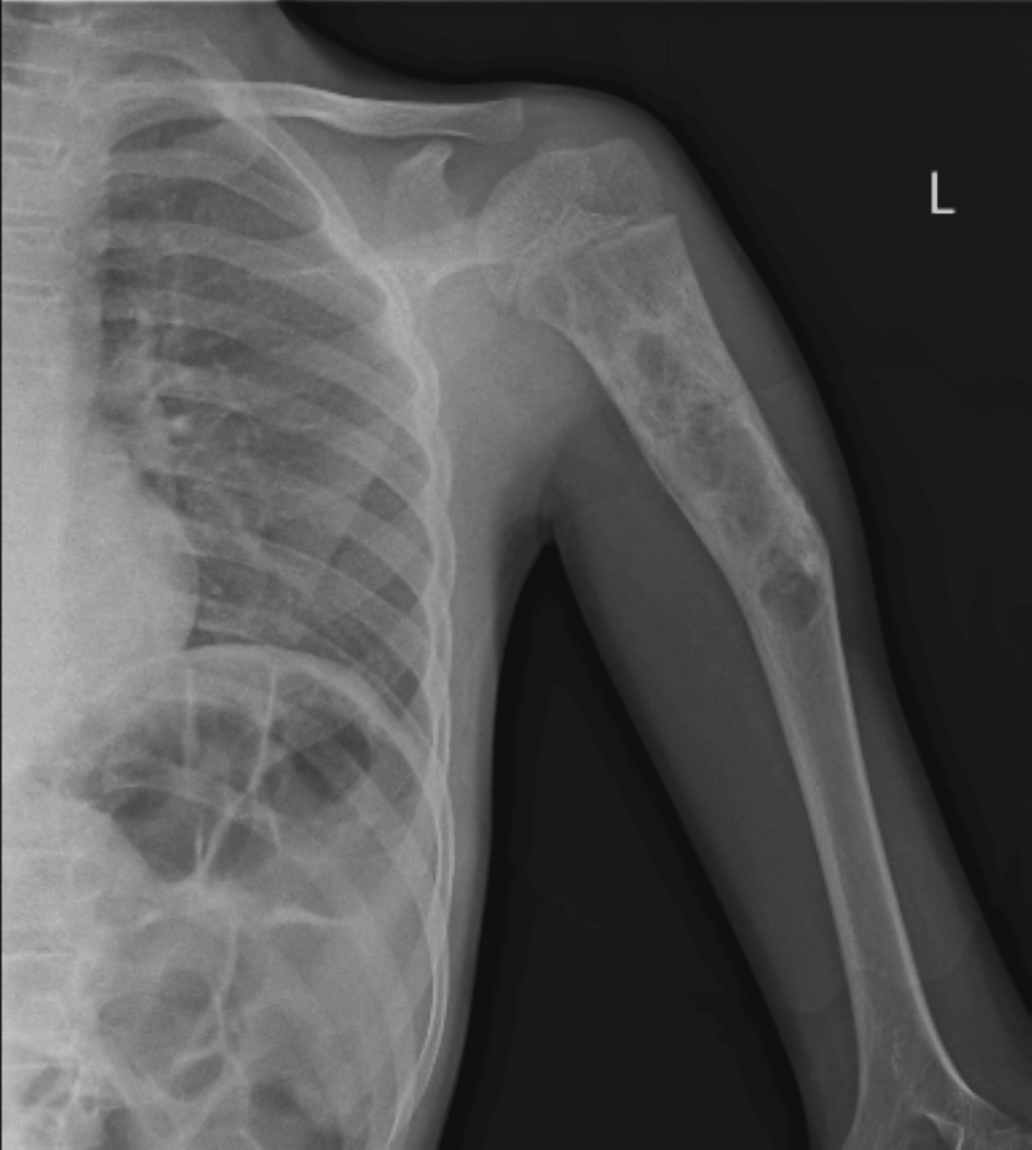
Humerus radiograph showing associated multiloculated expansile bone cyst in the left proximal humerus

The doctor advised an X-ray of the shoulder joint and humerus for radiological evaluation. The humerus radiograph revealed a well-defined, expansile, multiloculated cystic lesion in the proximal humerus involving the metaphyseal-diaphyseal junction, with a pathological fracture likely indicative of an SBC. No subluxation or dislocation was observed, as illustrated in Figure 1.

**Figure 2:**
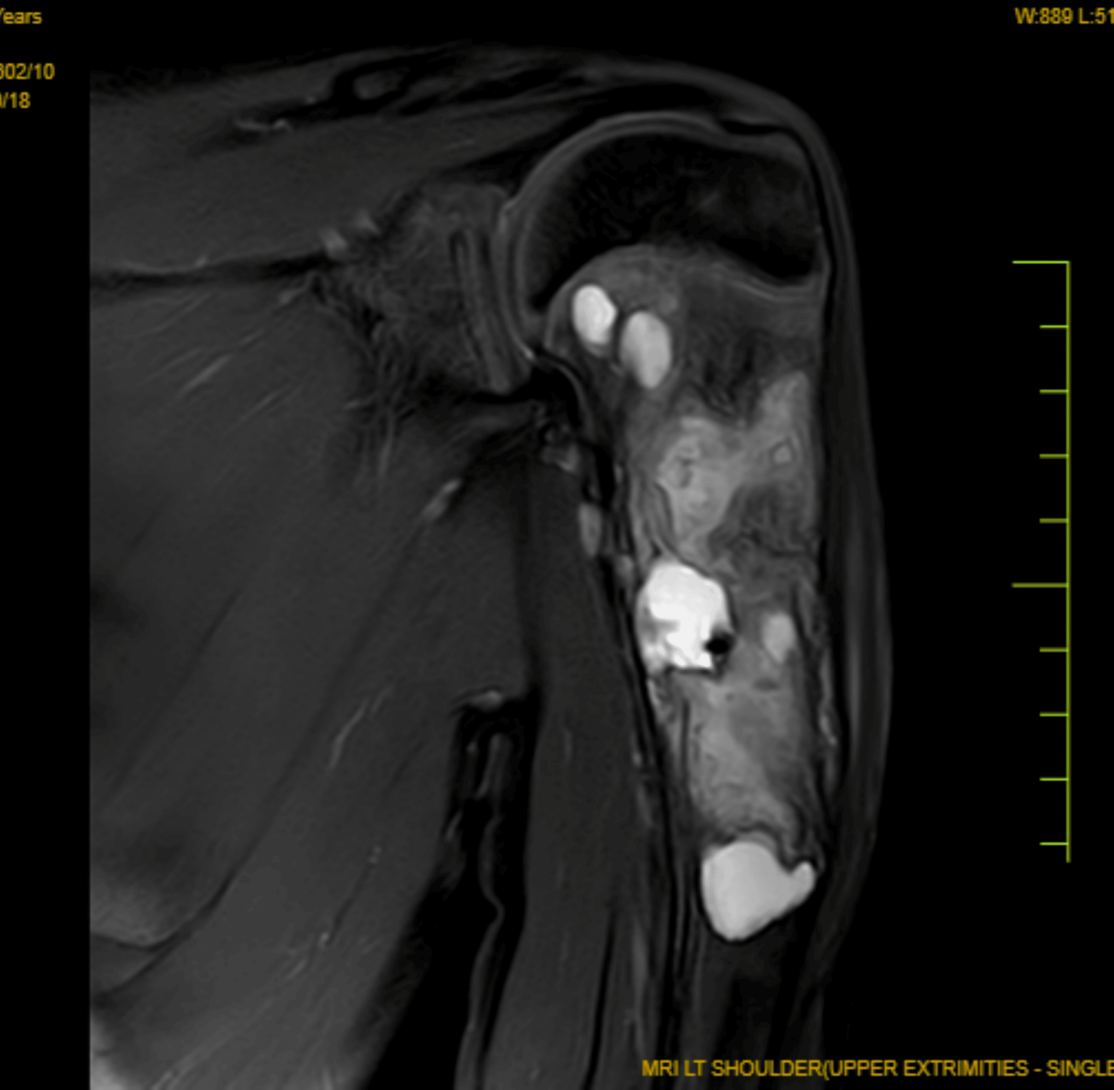
Coronal MRI showing intramedullary expansile lytic lesion in the proximal metadiaphysis of the humerus MRI: magnetic resonance imaging

The doctor recommended a magnetic resonance imaging (MRI) scan of the left humerus for further evaluation. The MRI report revealed an intramedullary expansile lytic lesion in the proximal metadiaphysis of the humerus, with a focal cortical break along the inferior aspect of the lesion and an adjacent periosteal reaction, consistent with an SBC with a pathological fracture. The shoulder joint, surrounding soft tissues, and muscles appeared normal, as illustrated in Figure 2.

Treatment

The patient underwent a comprehensive treatment regimen for a fracture and an SBC. The plan included a multivitamin syrup, 5 mL once daily; calcium carbonate syrup with added vitamin D3, 4 mL twice daily; and ferrous ascorbate syrup with added folic acid, 8 mL twice daily. Sclerotherapy was administered by injecting a chemical solution directly into the varicose vein, causing the vein walls to swell, adhere to each other, and seal shut, halting blood flow. As a result, the vein fades within a few weeks. Varicose veins are typically caused by weak or damaged valves within the veins. The patient's response to treatment was regularly monitored, with necessary adjustments made to the regimen.

Follow-up

Regular follow-up appointments were scheduled to monitor the treatment progress for the SBC and pathological fracture. Periodic tests, including complete blood count, liver function tests, renal function tests, and random blood sugar levels, were conducted, as shown in Table 2, to evaluate the effectiveness of the oral medications and make any necessary treatment adjustments. Additionally, an X-ray was advised every two months to monitor bone status and determine the need for further medication or additional sclerotherapy.

**Table 2:** The blood test report, including the reference range and units of all parameters

Parameter	Result	Reference range
Random blood sugar (mg/dL)	80	70-140
Complete blood count		
Hemoglobin (g/dL)	13.5	13.5-17.5
White blood cells (×10^3^/µL)	6.2	4.0-11.0
Platelets (×10^3^/µL)	250	150-450
Hematocrit (%)	41.0	38.8-50.0
Mean corpuscular volume (fL)	96.1	80-100
Mean corpuscular hemoglobin (pg)	30.0	27.5-33.2
Mean corpuscular hemoglobin concentration (g/dL)	34.5	32.0-36.0
Liver function tests		
Aspartate aminotransferase (U/L)	25	10-40
Alanine aminotransferase (U/L)	30	7-56
Alkaline phosphatase (U/L)	90	44-147
Total bilirubin (mg/dL)	0.9	0.1-1.2
Direct bilirubin (mg/dL)	0.2	0.0-0.3
Albumin (g/dL)	4.2	3.5-5.0
Total protein (g/dL)	7.0	6.0-8.3
Kidney function tests		
Blood urea nitrogen (mg/dL)	16	7-20
Creatinine (mg/dL)	1.2	0.6-1.2
BUN:creatinine ratio	16.7	10:20
Glomerular filtration rate (mL/min/1.73 m²)	90	>90

## Discussion

SBCs are not a rare condition, and the pathogenesis of this complication remains unclear. Cysts at the proximal end of the humerus are commonly treated with curettage, cauterization, and bone grafting. It has also been noted that premature epiphyseal closures, resulting in bone shortening, are a consequence of repeated pathological fractures, which can subsequently lead to growth arrest [1]. The cyst is irrigated to reduce bone-destructive enzymes such as gelatinase, remove the cyst membrane, and stimulate bone healing. Patients with bone cysts often show no change after the initial treatment and may require subsequent steroid injections to achieve results [2]. Open surgical procedures yield more successful outcomes, facilitating early recovery. New bone formation fills with a higher ratio of cystic cavities, eliminating the need for repeated surgeries and demonstrating a low recurrence rate and fewer pathological fractures. SBCs may occur in long bones, necessitating heightened vigilance for pathological fractures [5]. While the cavity may not have therapeutic implications for SBCs, accurate diagnosis and etiological assessment are crucial. As the lesion ages, the fluid in the cavity decreases and is eventually replaced by gas, promoting cyst growth [6].

The lesion and associated symptoms in the proximal humerus developed after physeal plate closure and were likely caused by repeated "kicks" during training sessions. This study describes an atypical SBC. Notably, a different radiographic image of the identical lesion appeared three years before its manifestation [7]. Many experts believed SBCs could be treated without additional intervention and healed before bone formation. Furthermore, risk factors such as pain, refracture, and deformity are significant. The primary goals in treating SBCs are to reduce the likelihood of pathological fractures, promote cyst healing, and alleviate pain. Various therapies for SBCs have been established, with open-operative approaches like curettage and bone grafting being the most used. These methods provide mechanical strength to ensure permanent attachment and preserve the bone’s blood supply [8].

Unicameral bone cysts (UBCs) have a varied natural history but often improve over time with growth. Some UBCs persist until maturity, while others resolve spontaneously during puberty. Despite a limited number of patients, this study found poor cyst healing following fractures in pathologic bone among the pubescent population. Results indicate a negative correlation between cyst healing after a fracture and normal growth promotion. To validate this hypothesis, long-term research with a diverse patient population is necessary [9]. Even with anticipated treatment, most SBC fractures heal. Neither surgical procedures nor steroid injections have a complete success rate. SBCs should be monitored only until symptomatic. If curettage is required, grafts or bone replacements should be employed. Uncomplicated fractures may benefit from intensive therapy to reduce the risk of recurrence [10].

## Conclusions

SBCs, also known as unicameral bone cysts, are benign, fluid-filled cavities typically found in the long bones of children and adolescents. While these cysts are often asymptomatic and discovered incidentally, they can occasionally cause pain or lead to fractures, especially in weight-bearing bones. Potential contributing factors include localized disturbances in bone growth or vascular anomalies. Diagnosis is typically confirmed through radiographic imaging, with X-ray and MRI revealing a well-defined, lytic lesion.

Treatment strategies vary based on the cyst’s size, location, and symptoms. Observation and regular monitoring may be sufficient for asymptomatic cases, while larger or symptomatic cysts may require interventions such as steroid injections or bone grafting. Overall, the prognosis for SBCs is favorable, as most respond well to treatment, enabling affected individuals to resume normal activities without long-term complications.
